# Baseline, Trend, and Normalization of Carcinoembryonic Antigen as Prognostic Factors in Epidermal Growth Factor Receptor-Mutant Nonsmall Cell Lung Cancer Patients Treated With First-Line Epidermal Growth Factor Receptor Tyrosine Kinase Inhibitors

**DOI:** 10.1097/MD.0000000000002239

**Published:** 2015-12-18

**Authors:** Yu-Mu Chen, Chien-Hao Lai, Huang-Chih Chang, Tung-Ying Chao, Chia-Cheng Tseng, Wen-Feng Fang, Chin-Chou Wang, Yu-Hsiu Chung, Kuo-Tung Huang, Hung-Cheng Chen, Ya-Chun Chang, Meng-Chih Lin

**Affiliations:** From the Division of Pulmonary and Critical Care Medicine, Department of Internal Medicine, Chang Gung Memorial Hospital-Kaohsiung Medical Center, Chang Gung University College of Medicine, Kaohsiung (YMC, CHL, HCC, TYC, CCT, WFF, CCW, YHC, KTH, HCC, YCC, MCL); and Department of Respiratory Care, Chang Gung Institute of Technology, Chiayi, Taiwan (WFF).

## Abstract

Among epidermal growth factor receptor (*EGFR*) mutation status unknown nonsmall cell lung cancer (NSCLC) patients, those with higher carcinoembryonic antigen (CEA) level are more likely to response to EGFR-tyrosine kinase inhibitors (TKIs) because they tend to have mutant epidermal growth factor receptor (*EGFR*). However, patients with higher CEA also have more tumor burden. With the above paradoxical evidence, it is prudent to understand the prognostic significance of baseline CEA in patients with *EGFR*-mutant NSCLC treated with first-line EGFR-TKIs. The clinical significance of the trend in CEA after treatment and the impact of CEA normalization during EGFR-TKI therapy are also unknown and potentially important.

A total of 241 patients who received first-line EGFR-TKIs were included. As to baseline CEA, patients were divided into normal, low, and high baseline CEA by cut point determined by receiver operating characteristic curves. As to CEA responses, patients were divided into 3 groups accordingly to their amount of CEA change after taking TKIs. In group A, 1-month follow-up CEA level decreased more than 35% with nadir CEA normalization; in group B, 1-month follow-up CEA level decreased more than 35% without nadir CEA normalization; and in group C, 1-month follow-up CEA level decreased less than 35% or increased.

Patients with higher baseline CEA levels had shorter progression-free survival (PFS) and overall survival (OS) (CEA > 32 vs 5–32 vs <5 ng/mL, PFS = 8.8 vs 11.3 vs 14.4 months, respectively, *P* < 0.001; OS = 17.8 vs 22.0 vs 27.9 months, respectively, *P* = 0.01). For trend and CEA normalization in groups A, B, and C, PFS was 14.3, 10.6, and 7.1 months, respectively (*P* < 0.001); OS was 29.7, 20.0, and 16.2 months, respectively (*P* < 0.001).

Baseline, trend, and normalization of CEA levels are potential prognostic markers for patients with *EGFR*-mutant advanced NSCLC treated with first line EGFR-TKIs.

## INTRODUCTION

The incidence of lung cancer is increasing in Taiwan, and it is the leading cause of cancer-related death worldwide.^1,2^ Asian lineage, never-smoker, and adenocarcinoma histology are well-known predictors of nonsmall cell lung cancer (NSCLC) patients harboring epidermal growth factor receptor (*EGFR*) mutations.^3–8^

In NSCLC patients harboring *EGFR* mutation, EGFR-tyrosine kinase inhibitors (TKIs) can improve quality of life, progression-free survival (PFS), and overall survival (OS).^9^ Several clinical parameters have been shown to affect the efficacy of EGFR-TKIs, including major mutation type, adenocarcinoma histology, tumor burden, Eastern Cooperative Oncology Group (ECOG) performance status (PS), baseline carcinoembryonic antigen (CEA) level, and lymphocyte-to-monocyte ratio.^10–17^

Previous studies in EGFR nonselective patients revealed that patients with a higher baseline CEA level are more likely to respond to EGFR-TKIs and have longer PFS.^13–15^ This phenomena may be attributed to a higher *EGFR* mutation rate in patients with higher CEA levels.^14,18^ However, previous studies also revealed that higher CEA level was correlated with higher tumor burden and more advanced stage.^19^

To the best of our knowledge, the prognostic significance of baseline CEA and the trend in CEA in patients with advanced-stag NSCLC with *EGFR* mutations who are treated with first-line EGFR-TKIs has not been well studied. In addition, the clinical significance of CEA levels normalization in CEA elevated patients during EGFR-TKIs therapies is not well known.

Therefore, we conducted a retrospective analysis to investigate the influence of baseline, trend, and normalization of CEA on clinical outcomes including PFS and OS in patients with NSCLC and *EGFR* mutation.

## MATERIAL AND METHODS

### Patient and Clinical Characteristics

From January 2011 to October 2013, this retrospective study was conducted at Chang Gung Memorial Hospital, Kaohsiung Medical Center in Taiwan. We included patients aged more than 18 years with pathologically (either histologically or cytologically) confirmed advanced stage, *EGFR*-mutant NSCLC who were receiving first-line EGFR-TKI. Patients who had previously received targeted therapy, chemotherapy, or immunological therapy were excluded.

Baseline assessments, including clinical characteristics, serum CEA, chest radiography and computed tomography (CT), brain magnetic resonance imaging, and bone scan were performed within 4 weeks before initiation of EGFR-TKIs.

Clinical characteristics were recorded including age, gender, diabetes mellitus (DM) history, smoking history, type of *EGFR* mutation, TNM status, number of distant metastases, and ECOG PS. Serial CEA data were collected if the patients’ baseline CEA level was ≥5 ng/mL. Trend of CEA level was obtained by dividing the 1-month CEA by the baseline CEA. CEA normalization was the lowest CEA among who had <5 ng/mL CEA levels during TKI therapy. The study was reviewed and approved by the Institutional Review Board of Chang Gung Memorial Hospital-Kaohsiung Medical Center, and informed consent was waived.

### Testing *of EGFR* Mutation

We obtained tumor specimens by CT-guided biopsy, bronchoscopy, pleural effusion cytology, or surgical biopsy. We used SCORPIONS and ARMS polymerase chain reaction (EGFR RGQ PCR Kit; Qiagen, Venlo, The Netherlands)^20^ for *EGFR* mutation analyses. We defined patients as having common mutations if they had pure exon 19 deletions or L858R mutations. Patients were defined as having uncommon mutations if they had mutations other than exon 19 deletions or L858R mutations or compound mutations.

### Response Evaluation *of EGFR*-TKI Treatment

For tumor response and disease status evaluation, patients underwent chest radiography at least once pre-month and chest CT every 2 to 3 months. Additional chest radiography and CT will be arranged whenever disease progression was suspected by clinician.

Disease status was evaluated using Response Evaluation Criteria in Solid Tumors criteria 1.1 by the clinician.^21^ PFS was defined as the time interval between the initiation of EGFR-TKIs administration and disease progression, death before disease progression, or the final visit before the end of follow-up.^16^ OS was defined as the time interval between the initiation of EGFR-TKIs administration and death, final visit before the end of follow-up or loss to follow-up.

### Statistical Analyses

Statistical analyses were performed using MedCalc (version 14.10.2). We used PFS longer or shorter than 12 months as binary variable for receiver operating characteristic (ROC) curves since median PFS in NSCLC patients harboring *EGFR* mutation treated with first line EGFR TKIs were 9.2 to 13.7 months in previous studies.^22–26^ ROC curves and Youden index were used to determine the optimal cut-off value for baseline and trend of CEA as prognostic factors. Univariable analysis of PFS and OS durations was performed using the Kaplan–Meier method and the log-rank test. Variables with *P* < 0.05 in univariable analysis were included into multivariable analysis using Cox proportional hazards regression test. The Kruskal–Wallis test was used for assessing the relationship between baseline CEA and TNM status as well as ECOG PS. A 2-sided *P* value less than 0.05 was considered statistically significant.

## RESULTS

### Patient Characteristics

**FIGURE 1:**
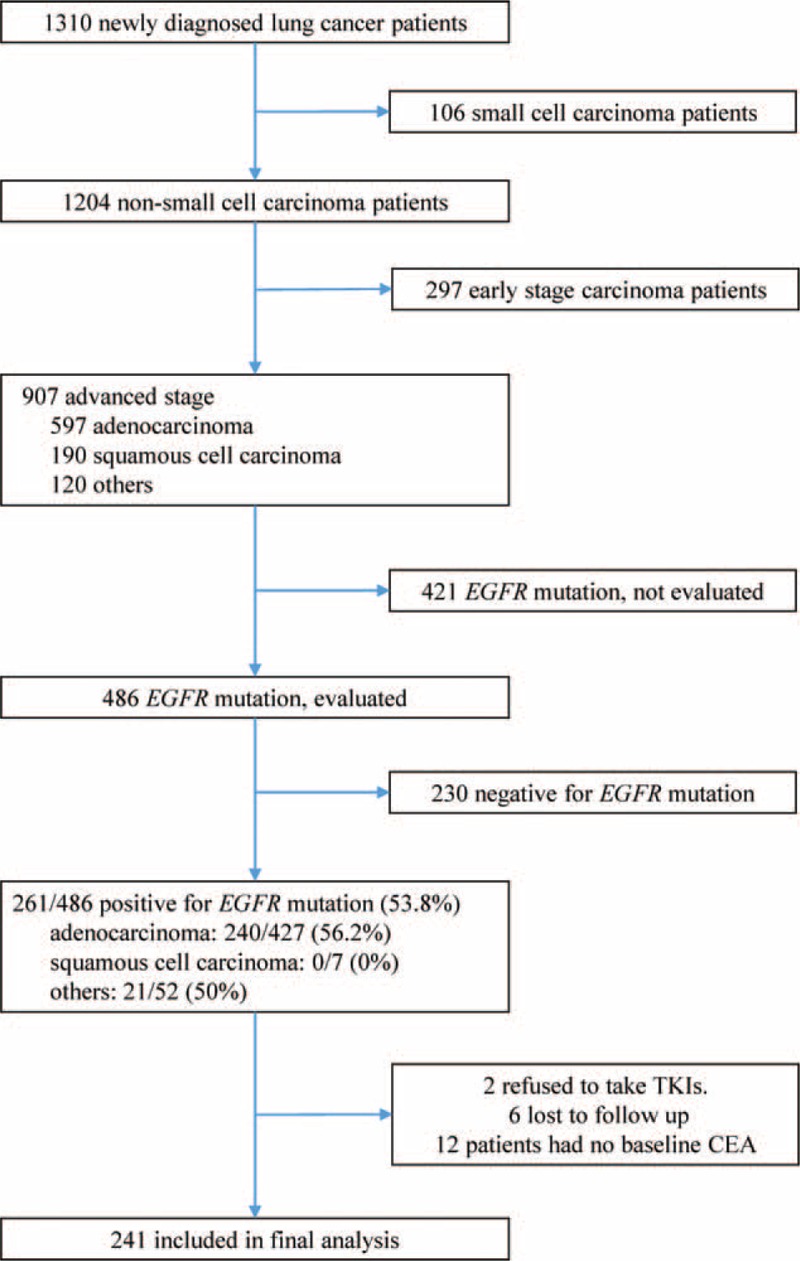
Inclusion, screening, and group assignment of patients. Among 1310 nonsmall cell lung cancer patients diagnosed between January 2011 and October 2013, 241 patients were included into final analysis.

Between January 2011 and October 2013, 1310 lung cancer patients were diagnosed (Fig. 1). Of 486 patients screened for *EGFR* mutations, 261 (53.7%) patients had *EGFR*-mutant NSCLC. Six patients were lost to follow-up, 2 patients refused to undergo treatment with TKIs, and 12 patients had no pretreatment serum CEA data. The final analysis data set consisted of 241 patients. The median follow-up time of study patients was 24 months, and the longest follow-up time was 45 months. The mean age of the study population was 64.9 years, median PFS was 10.3 months, and median OS was 22.0 months (Table 1). The baseline CEA was ≥5 ng/mL in 182 of 241 patients. Among these 182 patients, serial follow-up of CEA data were available for 130 patients. The baseline, follow-up, and nadir CEA levels are shown in Table 1. At the last follow-up, 205 (85.1%) patients showed disease progression and 129 (53.5%) had died. The best cut-off points for baseline and trend of CEA determined by ROC curve analysis were 32 ng/mL and 0.65 (35% decreasing from baseline), respectively. Based on above cut-off value for baseline CEA, patients were classified as high, low, and normal baseline CEA levels. Based on above cut-off value for trend of CEA, patients were classified as having a positive CEA response if their 1 month CEA decreased more than 35% compared with the baseline CEA, otherwise they were classified as no CEA response.

**TABLE 1:**
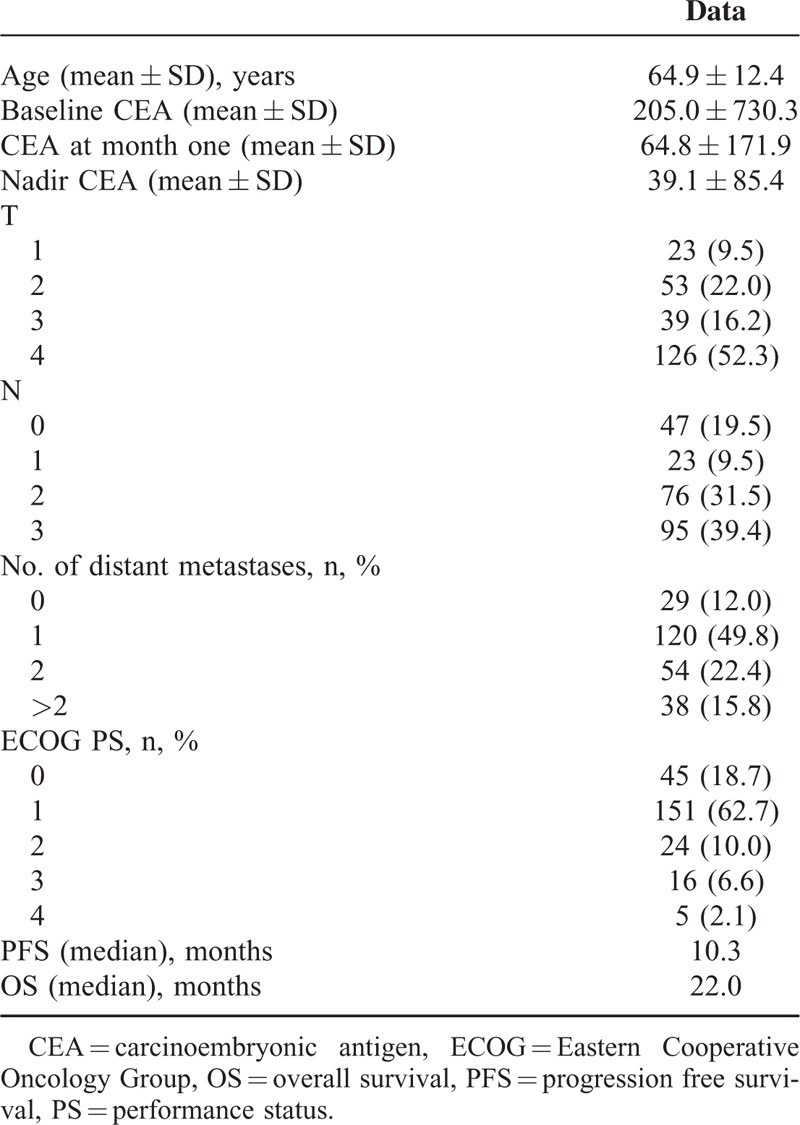
Clinical Characteristics of Patients

### Survival Analysis of Baseline Clinical Factors

**FIGURE 2:**
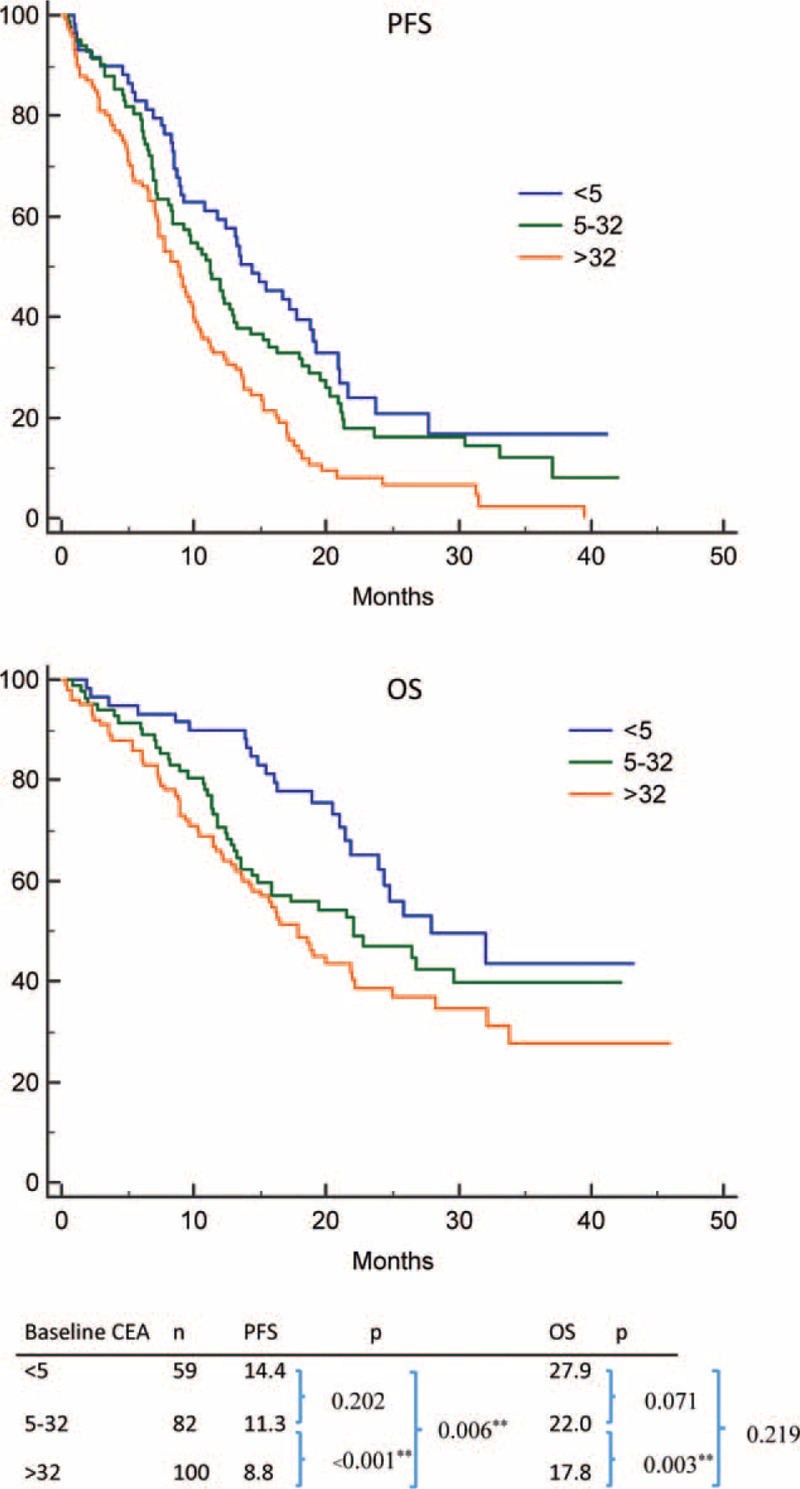
Impact of baseline carcinoembryonic antigen on epidermal growth factor receptor mutant nonsmall cell lung cancer patients treated with first-line tyrosine kinase inhibitors therapy. (Top) PFS between high, low, and normal baseline carcinoembryonic antigen patients; (bottom) OS between high, low, and normal baseline carcinoembryonic antigen patients. OS = overall survival, PFS = progression-free survival.

Patients with shorter PFS duration in the univariable analysis of clinical parameters included uncommon *EGFR* mutations (*P* < 0.001), higher baseline CEA (*P* < 0.001) (Fig. 2), more distant metastases (*P* < 0.001), and poor ECOG PS (*P* < 0.001) (Table 2). Age older or younger than 65 years, sex, history of DM, and smoking had no influence on PFS duration. Clinical predictive factors for a shorter PFS duration in multivariable analysis were uncommon *EGFR* mutations (HR 2.178, *P* = 0.001), baseline CEA > 32 ng/mL (HR 1.715, *P* = 0.005), more distant metastases (HR 1.928, *P* = 0.001), and poor ECOG PS (HR 1.709, *P* = 0.002) (Table 2).

**TABLE 2:**
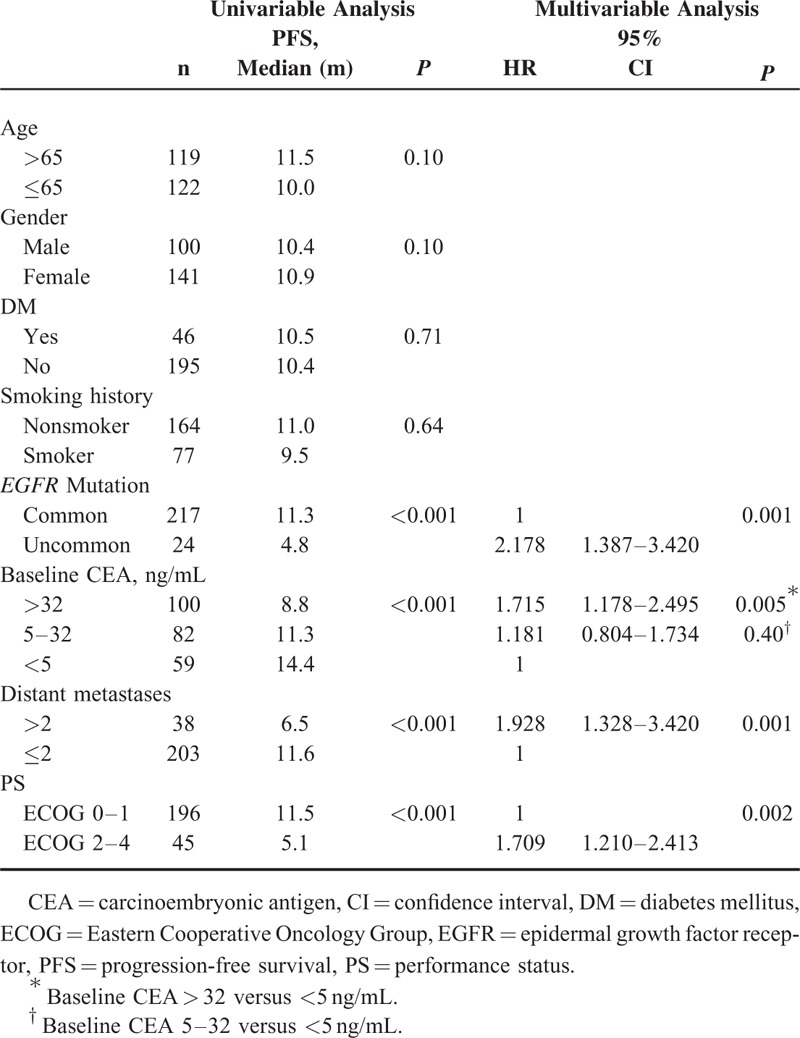
Univariable and Multivariable Analyses of Baseline Characteristics and Progression-Free Survival

Patients with shorter OS duration in the univariable analysis of clinical parameters included higher baseline CEA (*P* = 0.01), more distant metastases (*P* < 0.001), and poor ECOG PS (*P* < 0.001) (Table 3). Age older or younger than 65 years, sex, history of DM and smoking, and type of *EGFR* mutation had no influence on OS duration. Clinical predictive factors for a shorter OS duration in multivariable analysis included baseline CEA > 32 ng/mL (HR 1.718, *P* = 0.03), more distant metastases (HR 2.211, *P* < 0.001), and poor ECOG PS (HR 2.884, *P* < 0.001) (Table 3).

**TABLE 3:**
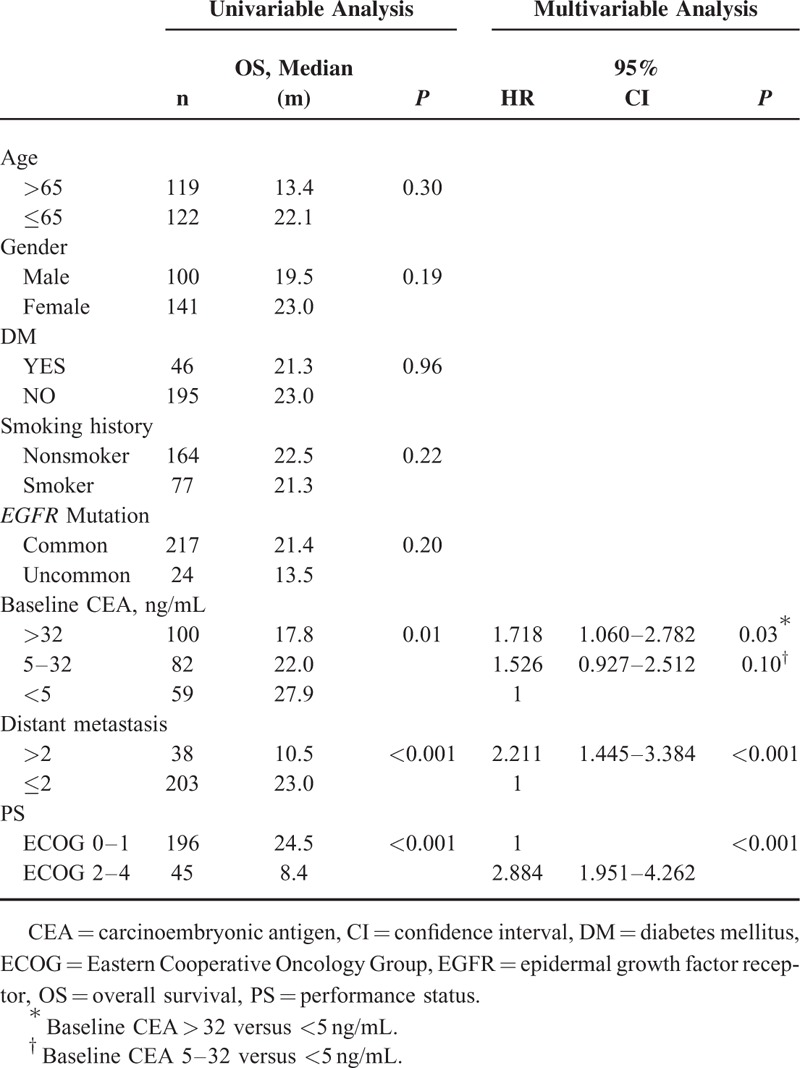
Univariable and Multivariable Analyses of Baseline Characteristics and Overall Survival

### CEA Response and Normalization

**FIGURE 3:**
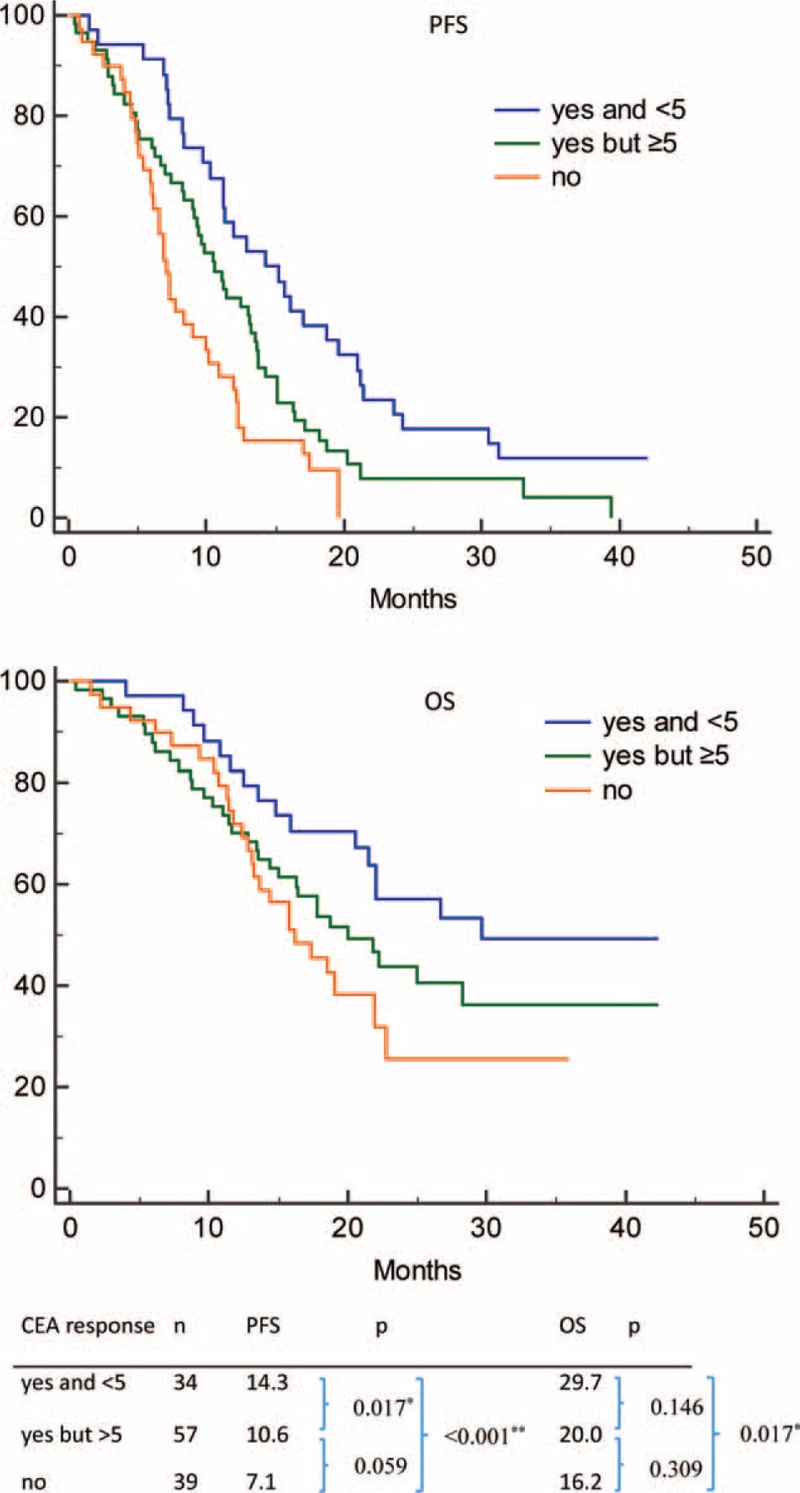
Impact of trend and normalization of CEA on epidermal growth factor receptor mutant nonsmall cell lung cancer patients treated with first-line tyrosine kinase inhibitors therapy. (Top) PFS in 3 subgroup patients: patients with both CEA response and normalization, with CEA response but without normalization, and in patients without CEA response; (bottom) OS in 3 subgroup patients mentioned before. CEA = carcinoembryonic antigen, OS = overall survival, PFS = progression-free survival.

**FIGURE 4:**
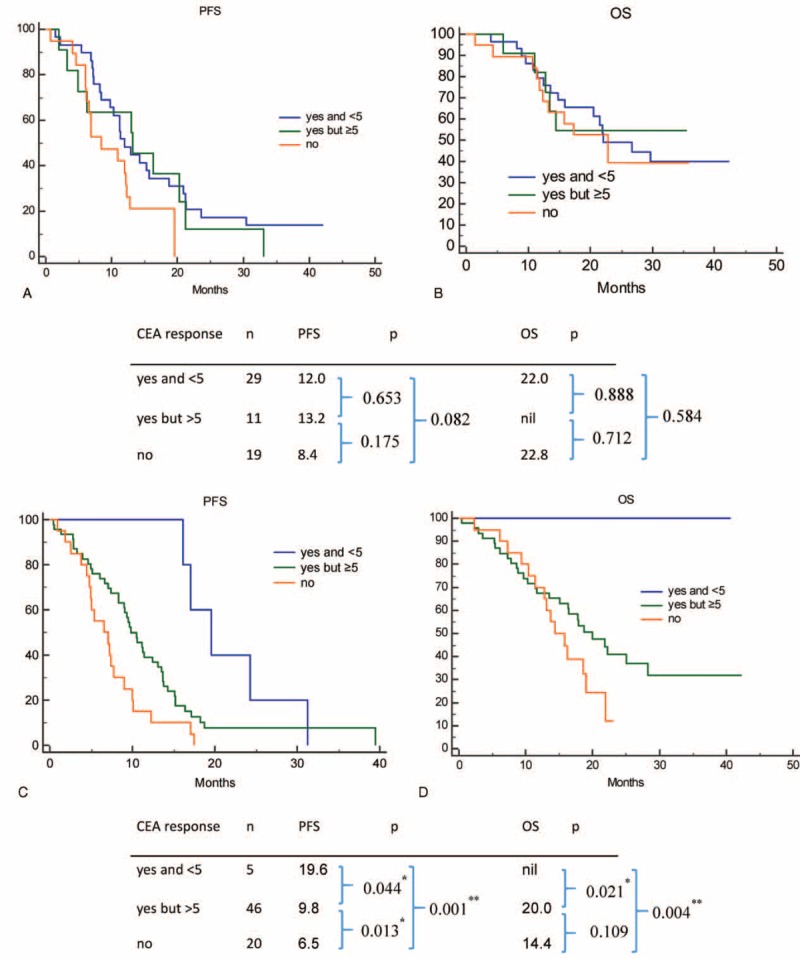
Impact of trend and normalization of CEA on patients with high or low baseline CEA among epidermal growth factor receptor mutant nonsmall cell lung cancer patients treated with first-line tyrosine kinase inhibitors therapy. Impact of trend and normalization of CEA on PFS (A) and OS (B) in patients with lower baseline CEA (baseline CEA 5–32); impact of trend and normalization of CEA on PFS (C) and OS (D) in patients with higher baseline CEA (baseline CEA > 32). CEA = carcinoembryonic antigen, OS = overall survival, PFS = progression-free survival.

Patients were divided into 3 groups accordingly to their CEA response (Fig. 3). In group A, patients had both a CEA response and normalization; in group B, patients had a CEA response and but had no CEA normalization; and in group C, patients were nonresponders. PFS duration was 14.3, 10.6, and 7.1 months in groups A, B, and C, respectively (*P* < 0.001). OS was 29.7, 20.0, and 16.2 months in groups A, B, and C, respectively (*P* < 0.001). We also further evaluated impact of baseline CEA on trend and normalization of CEA (Fig. 4). In patients with lower baseline CEA (baseline CEA: 5–32), trend and normalization of CEA had no significant prognostic effect for PFS (*P* = 0.166) and OS (*P* = 0.847). However, in patients with higher baseline CEA (CEA > 32), trend and normalization of CEA are prognostic factors for PFS (*P* = 0.002) and OS (*P* = 0.010).

### Association Between Baseline CEA and TNM or ECOG PS

Patients with higher CEA had more aggressive tumor behavior (*P* = 0.020) more lymph node involvement (*P* = 0.024) and more distant metastasis (*P* < 0.001) (Table 4). Patients with higher CEA also had a worse ECOG PS. In patients with PS 0, 1, 2, 3, and 4, the median CEA was 7.0, 27.0, 26.1, 55.1, and 57.0, respectively (*P* < 0.001).

**TABLE 4:**
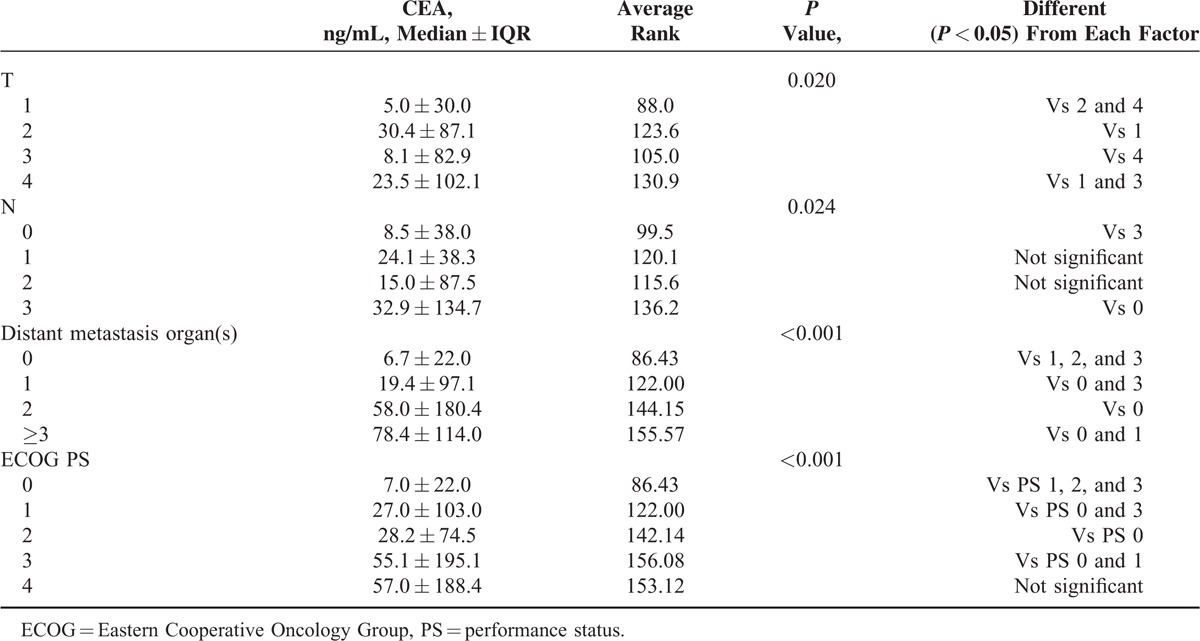
Association Between Pretreatment Carcinoembryonic Antigen Levels and Clinical Parameters

## DISCUSSION

CEA is a glycoprotein found in patients with carcinoma such as colon, rectum, stomach, pancreas, liver, and lung and in patients with inflammatory bowel disease.^27^ As a well-known tumor marker in colorectal cancer,^28^ role of CEA in lung cancer is still debated.

Our study demonstrated that baseline, trend, and normalization of CEA are potential prognostic factors in patients with NSCLC treated with first line EGFR-TKIs. Previous studies have revealed that higher baseline CEA level was associated with a worse prognosis in patients with early-stage gastric cancer, patients with lung cancer who underwent tumor resection,^29–31^ and in patients with advanced colorectal cancer treated with bevacizumab-based therapies.^32^ CEA has been reported to be crucial in colon cancer cells metastasis via cell adhesion to E- and L-selectin^33^ and correlated with higher tumor burden and more distant metastases.^34^ Paradoxically, however, previous studies in *EGFR* nonselective patients revealed that patients with higher CEA were more likely to response to EGFR-TKIs and have a better prognosis.^13–15^ Some believe that this discrepancy is because patients with higher CEA levels are more likely to have a positive *EGFR* mutation.^14,18^ After removing *EGFR* mutation status as a confounding factor, our study revealed that higher baseline CEA was associated with worse outcomes in *EGFR*-mutant patients treated with EGFR-TKIs, which was in line with study focus on colorectal cancer treated with bevacizumab-based therapies.^32^

Previous studies revealed that CEA response after operation and response to chemotherapy were prognostic factors in patients with NSCLC.^13,35,36^ Previous studies also revealed that normalization of CEA after surgery was a prognostic factor in patients with early-stage gastric, rectal, and lung cancer.^37–39^ However, the impact of CEA trend and normalization in patients treated with EGFR-TKIs is not well known. Our study revealed that CEA trend and normalization was a prognostic factor in *EGFR*-mutant patients treated with first line TKIs. However, this effect was only seen in patients with higher baseline CEA.

Our study had several limitations. First, we had no serial data of tumor burden, such as positron emission tomography metabolic tumor volume or total lesion glycolysis. Thus, the correlation between tumor burden and serum CEA level was not available. Second, we had no baseline and serial data of CYFRA 21-1, and neuron specific enolase, since recent studies revealed their prognostic effects in NSCLC patients.^40^ Thus, correlation between CEA, CYFRA 21-1, and neuron specific enolase became unavailable. Third, because our study was a retrospective study with a small patient population, a prospective trial is needed to validate these results.

In conclusion, out study revealed baseline, trend, and normalization of CEA are potential prognostic markers in *EGFR*-mutant NSCLC patients treated with first-line EGFR-TKI therapy.
